# High-Frequency Patterns in the Abundance of Benthic Species near a Cold-Seep – An Internet Operated Vehicle Application

**DOI:** 10.1371/journal.pone.0163808

**Published:** 2016-10-12

**Authors:** Damianos Chatzievangelou, Carolina Doya, Laurenz Thomsen, Autun Purser, Jacopo Aguzzi

**Affiliations:** 1 Jacobs University Bremen, Bremen, Germany; 2 Instituto de Ciencias del Mar (ICM-CSIC), Barcelona, Spain; 3 Alfred-Wegener-Institut (AWI), Bremerhaven, Germany; University of Ferrara, ITALY

## Abstract

Three benthic megafaunal species (i.e. sablefish *Anoplopoma fimbria*; pacific hagfish *Eptatretus stoutii* and a group of juvenile crabs) were tested for diel behavioral patterns at the methane hydrates site of Barkley Canyon (890 m depth), off Vancouver Island (BC, Canada). Fluctuations of animal counts in linear video-transects conducted with the Internet Operated Deep-Sea Crawler “Wally” in June, July and December of 2013, were used as proxy of population activity rhythms. Count time series and environmental parameters were analyzed under the hypothesis that the environmental conditioning of activity rhythms depends on the life habits of particular species (i.e. movement type and trophic level). Non-linear least squares modeling of biological time series revealed significant diel periods for sablefish in summer and for hagfish and crabs in December. Combined cross-correlation and redundancy (RDA) analyses showed strong relationships among environmental fluctuations and detected megafauna. In particular, sablefish presence during summer months was related to flow magnitude, while the activity of pacific hagfish and juvenile crabs in December correlated with change in chemical parameters (i.e. chlorophyll and oxygen concentrations, respectively). Waveform analyses of animal counts and environmental variables confirmed the phase delay during the 24 h cycle. The timing of detection of sablefish occurred under low flow velocities, a possible behavioral adaptation to the general hypoxic conditions. The proposed effect of chlorophyll concentrations on hagfish counts highlights the potential role of phytodetritus as an alternative food source for this opportunistic feeder. The juvenile crabs seemed to display a cryptic behavior, possibly to avoid predation, though this was suppressed when oxygen levels were at a minimum. Our results highlight the potential advantages such mobile observation platforms offer in multiparametric deep-sea monitoring in terms of both spatial and temporal resolution and add to the vastly understudied field of diel rhythms of deep-sea megafauna.

## 1. Introduction

The study of gas hydrates systems has gained progressive attention in both the geochemical and biological research fields, with the systems clearly showing the lack of homogeneity in the deep sea [1]. The potential exploitation of the hydrocarbon sources available at these sites generated further interest from an economic point of view, in relation to the quantity and quality of utilizable material, as well as their potential impact on the structural stability of the seabed [2–3]. Cold-seeps are considered biodiversity hotspots capable of sustaining a greater biomass than comparable areas of adjacent continental slopes, due to their high spatio-temporal habitat heterogeneity [4–6]. These systems may provide the principal energy pathways through bacterial chemosynthetic production [7–9]. Gas seepage, their characterizing element, can be affected by periodic bottom currents at scales of few hours (i.e. of semi-diurnal nature; [3]). Within this context, the importance of high-frequency, long-term and multidisciplinary ecological monitoring of such environments is evident.

Nowadays, the rapid technological advances in seabed exploration and monitoring techniques allow scientists have access to real-time biological and environmental data from various marine sites within the world ocean [10–13]. As described in [3, 14], an Internet Operated Deep-Sea Crawler (Wally) has been deployed at the gas hydrates site in Barkley Canyon (NE Pacific, Canada) and in operation since September of 2010, as a part of the NEPTUNE Canada Cabled Observatory network (www.oceannetworks.ca). The uniqueness of this monitoring platform lies in the combination of mobility with the high-frequency acquisition of multiparametric data throughout long-term deployment, whilst remaining in communication with land-based researchers 24/7 *via* the Internet. Until now all these features could be found distributed among the previously existing mobile and fixed benthic technologies (i.e. ROVs, AUVs, and landers *versus* cabled observatory nodes), rather than on one mobile platform.

Remote, high-frequency and long-lasting image monitoring of the continental margin and abyssal communities is nowadays increasingly required in order to link measurable biodiversity to species turnover as a product of activity rhythms [15–17]. Animal activity patterns may come as a response to a persistent temporal structure within an environment (i.e. geophysical cycles in light intensity and internal tides), resulting in 4 behaviorally different phenotypes, as identified in laboratory controlled conditions: diurnal, nocturnal, crepuscular and arrhythmic animal behaviors [18–21]. Species use time as an ecological resource while they are competing with others of a partially overlapping habitat niche, forming the dynamics of predator-prey relationships and finally in behavioral adaptations that come as a response to their own physiological limitations [22–24]. In benthic communities, the “zeitgeber” (the environmental cue forcing animals to keep their internal clock synchronized) can be either sunlight or periodic hydrodynamism (e.g. current velocity and direction, internal tides) [25–27]. Intraspecific plasticity of activity patterns is often observed [28], as local variability of the aforementioned variables may lead to distinct populations facing different conditions. On continental margins, animal response to such triggers is commonly expressed as diel displacement, either vertically, horizontally or in and out of the substrate (i.e. pelagic migrations, nektobenthic or endobenthic movements) [15]. Apart from regulating the dynamics of benthic communities, vertical migrations (benthopelagic coupling) can affect the redistribution of nutrients and organic matter in the ocean [29–30]. Additionally, periodical change in local abundances of benthic species can affect their detection in a certain space and time, leading to potential bias in traditional scientific sampling by trawling and affecting commercial fishing activities [31–32].

In this study, crawler imaging and environmental data were used to identify any diel patterns within the visual counts of the most abundant megabenthos at the Barkley Canyon gas hydrates site, and whether they exhibited a relationship with concomitant environmental forcing. We hypothesized that the activity of megafauna with different ecological traits in relation to motility and trophic level, is modulated by different environmental variables.

## 2. Material and Methods

### 2.1 Study Site and Data Acquisition

**Fig 1:**
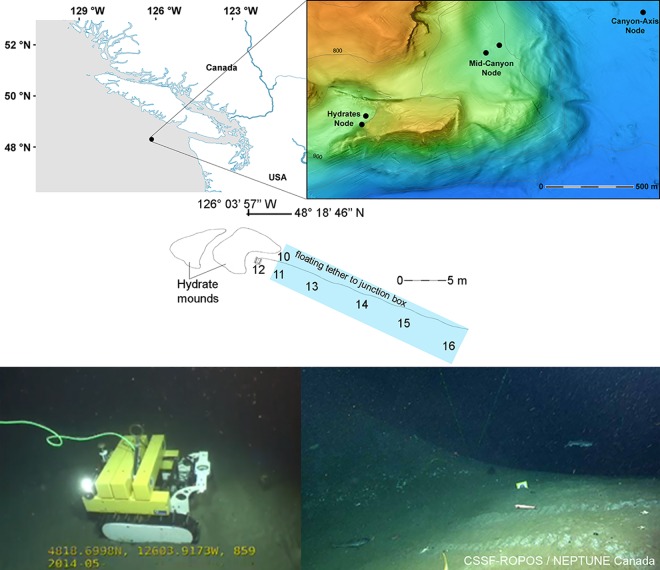
Map of the study site. The position of the gas hydrates site in Barkley Canyon, off Vancouver Island, and the path followed by Wally when the linear transects were performed (i.e. between waypoints 16–10). Canyon map modified from [34]. Transect representation modified from [14].

The study was conducted at the gas hydrates site of NEPTUNE Cabled Observatory network (www.oceannetworks.ca), located on a small (1 Km^2^) plateau in Barkley Canyon (Fig 1; 48° 18’ 46” N, 126° 03’ 57” W), at approx. 890 m depth. Authorization for conducting research was provided by the Transport Canada (www.tc.gc.ca/), after Fisheries and Oceans Canada (http://www.dfo-mpo.gc.ca/) assessed that the cable installation would not have a negative impact on fish habitat.

Oceanographic conditions in Barkley Canyon at these depths are known to differ between seasons, with distinct water flow, temperature and chlorophyll flux patterns previously reported for summer and winter months [3, 14, 33]. Following [33], the annual cycle was divided into two main seasons (summer and winter). The months analyzed in this current study are June, July and December of 2013, since they were deemed as a sampling period representative of the oceanographic conditions and community structure of the location and also provided material that matched the study’s criteria (see Section 2.2).

Linear, constant back and forth imaging census transects of 20 m length (i.e. between waypoints 16 and 10; Fig 1) were carried out with Wally every 4 hours during 5 days, in the first week of each calendar month. All transects were video-recorded with a Panasonic web camera (BB-HCM580; 720 x 480 pixel) mounted in a glass globe designed to resist high pressure levels up to 6000 m depth [14]. Driving and recording were stopped by the researcher operating the crawler every time a sediment resuspension event was impeding visibility (i.e. < 70% of the Region of Interest visible). All video footage is archived and can be viewed online in the Ocean Networks Canada database (dmas.uvic.ca/).

Water flow and temperature were measured with an ADCP 2 MHz (Nortek Aquadopp Profiler AQD 9917) fixed on the crawler. Oxygen concentration measurements were taken using a Sea-Bird SBE 63 Dissolved Oxygen Sensor (Device Code 630109) deployed at the hydrates site. Density data were provided by the CTD Sea-Bird SeaCAT SBE16plus V2 7027 deployed at the POD4 site (mid-canyon east, at 896 m depth). Finally, the sensor used to measure chlorophyll levels was an ECO-FL-NTU (RT) 2973 (WET Laboratories), deployed also at POD4.

### 2.2 Data processing

Visual count variations can be considered as a direct proxy of populational behavioral rhythms, with animal rate of displacement controlling their chance to be spotted in the camera’s field of view during each sampling window [26–27, 35].

Stationary observations of the seafloor and video sequences with low visibility were discarded and the remaining video duration was further used. Pictured animals were classified to the lowest possible taxonomic level following [36] and then counted. Since there was no information available on the exact surface covered in each transect, animal counts data were summed for each pair of transects (i.e. back and forth between waypoints) and then normalized to number of individuals per 10 min. This normalization was based on the assumption that the differences in surface covered during the video transects were minimal and the underlying error was constant, leading to no effect on the final outcome. As a result, the biological time series consisted of a total of 92 observations taken at a 4 h frequency, each of these being the normalized sum of 2 transects. In total, over 21 h of footage were considered to be of acceptable quality for the faunal analysis.

**Fig 2:**
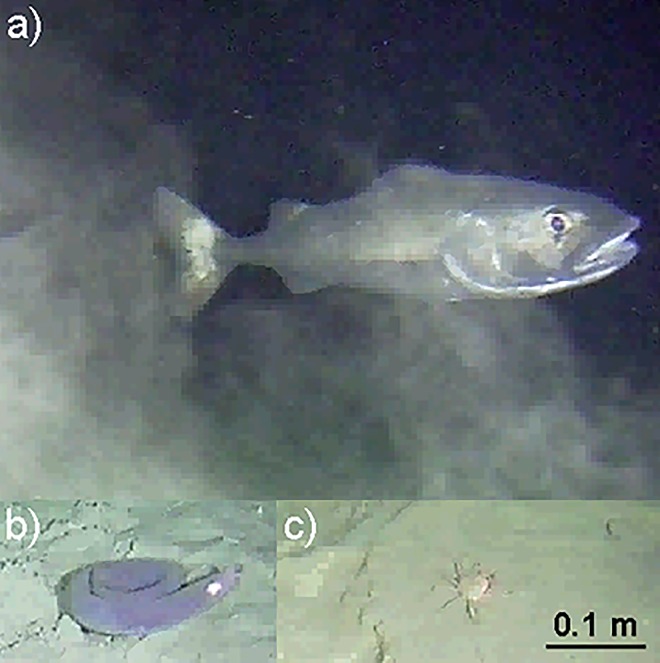
Photos of the studied species. The three species studied, as recorded by Wally in transects at the hydrates site; a) *A*. *fimbria* b) *E*. *stoutii* and c) juvenile crab. The same scale bar applies to all 3 plates.

For the statistical analyses, species time series containing 0 (zero) counts in more than 1/3 of the observations were discarded from further analyses to prevent misleading results, as it would be extremely hard to detect periodicities with statistical significance and low bias in short, zero-inflated time series [15]. As a result, the three most abundant and regularly spotted species that were subsequently studied were the sablefish *Anoplopoma fimbria* (can also be found by the name black cod) in June and July, the pacific hagfish *Eptatretus stoutii* in December and a group of juvenile crabs in the same month, which the authors believe are grooved tanner crabs, *Chionoecetes tanneri* (Fig 2). The small size of specimens and the regularly turbid conditions at the site did not permit a 100% precise identification, so hereafter they will be referred to as a juvenile functional group and treated as such. A complete species list, along with a table containing the abundances of all the observed species is provided in [37].

Environmental data were downloaded from the Ocean Networks Canada database (dmas.uvic.ca/) and averaged into 1 h bins for high resolution and flexibility in data treatment. This step was performed with MATLAB R2014b [38]. Current velocity data that were initially provided in the form of 2 components-vectors (North-South and East-West) were transformed to flow magnitude and flow direction with the use of the package “circular” [39] of the R statistical language [40]. These two variables were later treated with the R package “plotrix” [41] to construct polar plots, which allowed the visual assessment of water flow variability. All environmental data were subsequently averaged by 4 h using the R package “zoo” [42] and used in cross-correlations and RDA analyses with visual count time series (see Section 2.4). Hourly environmental data are provided in S1 Table. Visual counts and environmental data at 4 h frequency are provided in S2 Table.

### 2.3 Periodogram analysis and modeling

We carried out an implemented periodogram analysis in order to match the peculiar features of the crawler’s video-censusing frequency and duration. To tackle this methodological challenge, we customized a statistical approach based on non-linear least squares models. Accordingly, faunal data were plotted against time (i.e. normalized abundances as recorded every 4 h) and non-linear (sinusoidal) curves were fitted to each time series with R statistical language [40], as a commonly accepted alternative to traditional time series analyses for the detection of biological rhythms [43]. In this way, the best approximations of the underlying periods in visual counts were obtained. All models follow Eq 1.1, with a linear ascending trend added in the case of sablefish (Eq 1.2).

$$\alpha_{1}\;\cos\left(2\pi t/\alpha_{4}\right)+\alpha_{2}\;\sin\left(2\pi t/\alpha_{4}\right)+\alpha_{3}$$ (1.1)

$$\alpha_{1}\;\cos\left(2\pi t/\alpha_{4}\right)+\alpha_{2}\;\sin\left(2\pi t/\alpha_{4}\right)+\alpha_{3}+\alpha_{5}t$$ (1.2)

Where t is an integer [1:n] representing a time step of 4 h and a_4_ is the corresponding period.

We screened for significant periodicities in oceanographic parameters, which were later coupled to the visual counts of the different megafaunal species and used as a proxy of environmental modulation on animal behavior. Lomb-Scargle periodogram analyses were applied on hourly datasets using the “lomb” R package [44]. In output plots, the periodicity is indicated as the peak of this statistic’s power that crossed the significant threshold set for α = 0.05.

### 2.4 Cross-correlations and RDAs

Cross-correlation analyses were performed in R statistical language [40] between biological and environmental (i.e. normalized abundances as recorded every 4 h and 4 h averages respectively) time series, in order to reveal the significant (α = 0.05) response of animals to the fluctuations of oceanographic parameters. As this is not a two-way process (i.e. animal abundances do not affect the oceanographic parameters tested), negative lags are not presented. The power of the statistic is presented at normalized scale (i.e. from -1 to 1). Since cross-correlations were used as a complementary method to interpret periodicity in megafaunal abundances, oceanographic parameters that did not display periodic signal were not tested.

Furthermore, the overall effects of environmental parameters on animal abundances were tested with Redundancy Analysis (RDA) performed in the “vegan” R package [45], for each of the three months studied. Based on the results of the cross-correlations (see 3.2), the biological data input for the RDAs was lagged for 12 h. Environmental data were centered and scaled (i.e. use of z-scores of the 4 h averages as, input instead of raw values) prior to analyses. The output is presented as biplots, an advanced version of scatterplots that depicts ordination (here expressed as Euclidean distance) of both observations and variables.

### 2.5 Waveforms

The 4 h sampling frequency for biological data allowed the creation of 6 bins per day, with each bin containing the averaged corresponding value across the 5 sampled days of each month. The obtained waveform curves for each species were plotted together with a horizontal threshold line, the Midline Estimated Statistic Of Rhythm (MESOR; see [46]). Values above this line represented the peak, hence describing the temporal amplitude of biological data. The MESOR was computed by re-averaging all curve values. Waveform curves were also calculated for the hourly environmental data and added onto the output plots when a significant correlation between the abundance of a species and an environmental variable was indicated.

## 3. Results

### 3.1 Assessment of periodicities

**Fig 3:**
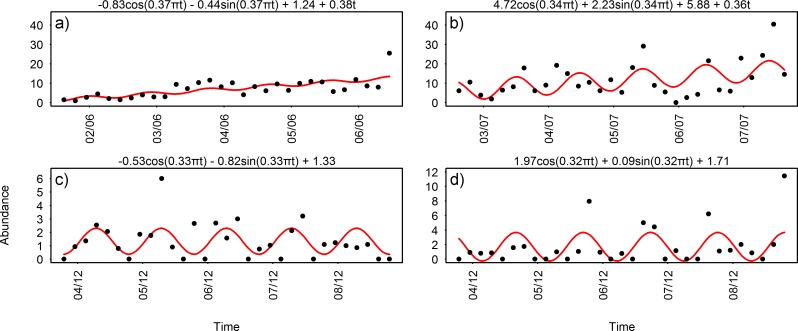
Non-linear least squares models of biological time series. Sinusoidal curves (red) fitted to the animal counts series and their corresponded equations with calculated coefficients; a) and b) *A*. *fimbria* in June and July respectively, c) *E*. *stoutii* in December and finally, d) juvenile crabs in December. Black points represent animal abundances for each pair of transects (i.e. back and forth, 4 h frequency). Note the difference of scale among species in the number of counts, as well as the ascending trend in sablefish counts (see Section 4.1).

The sinusoidal models of biological data (Fig 3) revealed significant diel periodicities in the number of animal counts, close to the 24 h period. The periods varied from 21.62 h for the sablefish in June, to 25 h for juvenile crabs in December. The shortest periodicity could be a product of the scattered sampling frequency (i.e. 4 h) and of the reduced length of the time series, and should be interpreted together with the results of the cross-correlation analysis (see below). A graphic representation of the model residuals (i.e. plots of residuals vs fitted values and histograms of residuals) is provided in S1 Fig.

**Fig 4:**
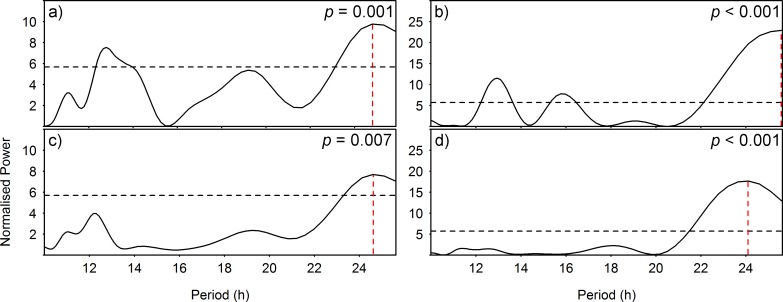
Periodograms of environmental parameters. Lomb-Scargle periodograms for: a) and b) flow magnitude in June and July respectively, c) chlorophyll concentration in December and finally, d) oxygen concentration in December. The horizontal dashed lines indicate significance for α = 0.05. The *p values* correspond to the peak in each periodogram (i.e. red, vertical dashed lines).

The periodogram analysis output for hourly environmental data (Fig 4) showed the presence of significant rhythmicity in flow magnitude and the concentrations of chlorophyll and oxygen. In all 4 cases the dominant period was of diel character (i.e. P = 24.79 h, 25.63 h, 24.60 h and finally, 24.12 h for plates a) to d) respectively), with shorter periods also present in flow data, indicating distinct tidal signals.

### 3.2 Response to environmental fluctuations

**Fig 5:**
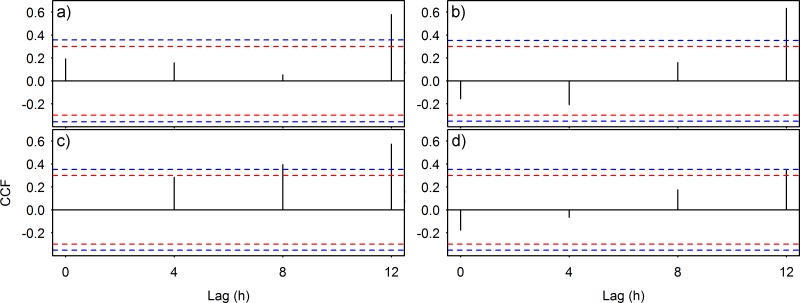
Cross-correlations between animal counts and environmental parameters. Analyses used to assess the presence of temporal lag in the response of animal behavior to environmental fluctuations; a) and b) *A*. *fimbria* and flow magnitude in June and July respectively, c) *E*. *stoutii* and chlorophyll concentration in December and finally, d) juvenile crabs and oxygen concentration in December. “Cross-Correlation Function, (CCF)” is the measure of the power of the statistic. The dashed blue lines represent a CCF significance for α = 0.05 (critical values: ±1.96/√n = approx. ±0.36 in a, and ±0.35 in b, c and d, where n is the sample size). The dashed red lines represent a CCF significance for α = 0.1 (accordingly, critical values: ±1.65/√n = approx. ±0.3 in all four plates).

Significant correlations between biological and 4 h averaged environmental time series are presented in Fig 5. In the first three cases, sablefish and hagfish responded positively to environmental variations (i.e. power of ~0.6 against flow magnitude and chlorophyll concentration, respectively) after 12 h for a level of significance α = 0.05, while juvenile crabs had a similar response (i.e. power ~0.4 to changes in oxygen concentration) for α = 0.1.

**Fig 6:**
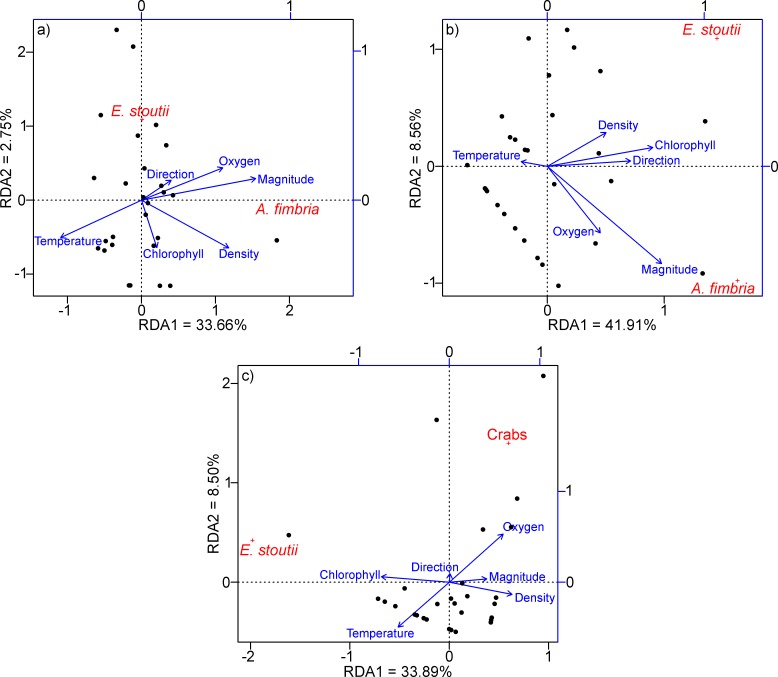
Redundancy Analysis (RDA) of the hydrates benthic community. RDA biplots for each one of the three months of the study period; a) June, b) July and c) December. The direction and length of the blue vectors depicting environmental variables represent positive or negative correlation with the ordination and proportionality of correlation respectively. Their biplot scores are depicted on the blue axes (normalized range -1 to 1). The scores for sites and species are weighted for scaling reasons and depicted on the black axes.

The RDA biplots (Fig 6) between environmental parameters at t and biological data at t + 12 h provided a general view of the lagged effect of environmental variations on the species studied. Regarding the species that displayed periodicities in their abundances, sablefish numbers appeared to be strongly related to flow magnitude. In a more detailed view, this species scores were higher for the RDA1 axis than for the RDA2 axis (i.e. 1.56 vs ~0 in June and 1.31 vs -0.53 in July). In both months, the stronger vector on the RDA1 was flow magnitude (i.e. scores 0.77 in June and 0.73 in July). On the other hand, the pacific hagfish and juvenile crab abundances in December were influenced by chlorophyll and oxygen concentrations, respectively. In this month, the hagfish score was higher for the RDA1 axis than for the RDA2 axis (i.e. -1.51 vs 0.23), while the opposite was observed for the crabs (i.e. 0.46 vs 0.76). The dominant vectors in each axis were chlorophyll for RDA1 (i.e. score -0.75) and oxygen for RDA2 (i.e. score 0.53). For detailed RDA scores for species and environmental parameters see S3 Table.

**Fig 7:**
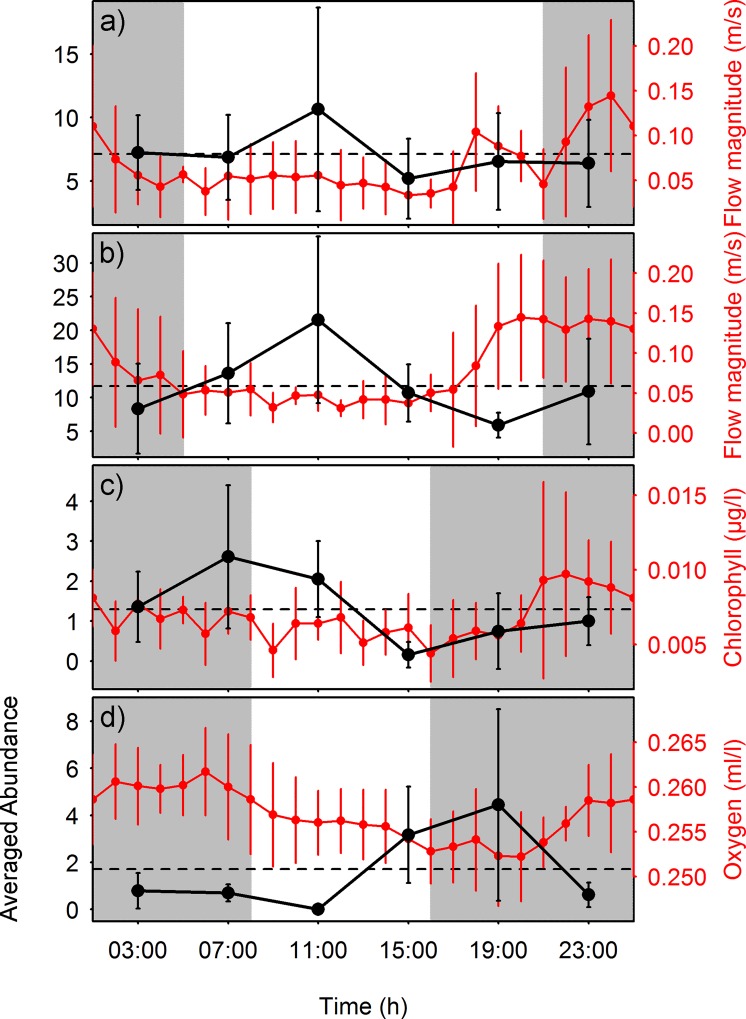
Waveform analyses of concomitant biological and environmental data. Waveforms of animal counts (black, 4 h sampling frequency) as the average (±sd) of the 5 days sampling period and of environmental parameters (red, hourly); a) and b) *A*. *fimbria* in June and July respectively, c) *E*. *stoutii* in December and finally, d) juvenile crabs in December. Note the difference in scale among different species. The dashed horizontal lines correspond to the MESOR. Shaded parts represent the approximate night duration at the time of the study. *X axis* values correspond to local time (PST).

As displayed visually in the output of waveform analysis (Fig 7), sablefish counts during the summer months peaked (i.e. mean values above the MESOR) right before midday, at a time of low currents. In contrast, hagfish were more abundant at crepuscular hours (dawn), following a peak of chlorophyll during the night. Finally, juvenile crab abundance reached a maximum in the early night hours, when oxygen concentrations were close to their minimum. The peaks in the waveforms of these two species come with a phase difference of 12 h over the same study period (i.e. hagfish and crabs in December of 2013). The waveform curves for visual count time series presented high standard deviation at their respective peak points. Nevertheless, the curve shape remains the same when the waveforms are calculated excluding the maximum values for each time bin (not shown), which reduces the possibility of biased/misleading results.

Table 1 gives a summary of the characteristics of species rhythmic activity and the environmental parameters which correlate with their abundances and life habits (i.e. swimming in the water column for sablefish and close relationship to the seabed for hagfish and juvenile crabs).

**Table 1:** Species life habits (i.e. SW for swimmer, WL for walker, NKB for nektobenthic, ENB for endobenthic and EPB for epibenthic) in relation to their mode of displacement, the summary of their periodic activity (i.e. D for diurnal, C for crepuscular and N for nocturnal) and the most important environmental variables that regulate it.

Species	Period—Coefficient a_4_ *p value*		Phase	Movement Type	Env. Variable
*A*. *fimbria*	21.62	<0.001	D	SW—NKB	Flow magnitude
	23.53	<0.001	D		Flow magnitude
*E*. *stoutii*	25.24	<0.001	C	SW—ENB	Chlorophyll
Crabs	25	<0.001	N	WL—EPB	Oxygen

## 4. Discussion

The major objective of the current study was to test for the presence of diel patterns in visual counts of the most abundant and regularly recorded species in video transects performed with Wally at the methane hydrates site of Barkley Canyon. We established a relationship between animal activity rhythms and the environmental parameters that could substitute light intensity as the main “zeitgeber” at such depths, according to different species ecological habits. In the aphotic deep-sea, diel rhythms are a product of cycles mainly triggered by oceanographic processes, varying locally and regionally. As a result, our knowledge on such patterns remains limited, and generalizations should be treated cautiously. Anyway, present results fall within the expected scenarios on the expression of diel activity at such depths, with animals entering and leaving the field of view as they move on a daily basis in the water column or on the seafloor [15, 17, 28].

All targeted species showed a similar, near 24 h periodicity (see Table 1), as indicated directly by the least squares models calculated (see Fig 3) and indirectly by the cross-correlations (see Fig 5) between sablefish and flow magnitude, hagfish and chlorophyll and finally, juvenile crabs and oxygen concentrations respectively, also showing diel fluctuations (see Fig 4). The combined outcome of these analyses revealed a 12 h lagged response of the animals to such environmental parameters, depending on their ecology. Species inhabiting the benthic boundary layer with different locomotion strategies can react differently to changes in hydrodynamic conditions, with animals in closer relation to the seafloor being less susceptible to flow variations than swimmers [26, 47]. Accordingly, sablefish swimmers previously reported performing nektobenthic movements within the canyon (see Section 4.1), were highly affected by diel variation of flow magnitude. On the other hand, the two species that are more closely associated to the seabed (i.e. the endobenthic burrower hagfish and the epibenthic crabs; see Sections 4.2 and 4.3 respectively) were less affected by currents but responded primarily to diel changes in the concentrations of chlorophyll and oxygen. This was also confirmed by the RDA analyses (see Fig 6), where flow magnitude and chlorophyll and oxygen concentrations were the variables most closely associated with the respective sablefish, hagfish and crab abundances after a 12 h lag. Since no reverse flow conditions or major stochastic events occurred during the study period (see S2 Fig), our results should be considered an accurate representation of the community under normal oceanographic conditions.

### 4.1 Activity rhythms of sablefish

Sablefish activity in both summer months was connected to flow magnitude, with a peak right before midday (i.e. 11:00 local time PST; see Fig 7). This coincided with a time of low flow, which reached maximum velocity at night. A similar pattern was found at a nearby site [27], with most individuals recorded during times of low currents, as previously reported for the ecologically similar Atlantic cod *Gadus morhua* [48]. Periodic changes of the current regime in deep-sea environments can be detected by fish *via* their sensitive, well-developed lateral line systems, enabling them to keep their activity patterns synchronized [25]. Another study at similar depths in Barkley canyon reported the relationship between sablefish activity and tidal phenomena, this time expressed as water density [35]. In accordance, we believe that the ascending trend in sablefish counts in June and July of 2013 is a reflection of a lower frequency tidal signal (i.e. lunar tides), since the 5 days sampling periods for both months were entirely integrated in the spring tide phase (i.e. towards new moon).

Sablefish found at these depths are most commonly adult predators with demersal life habits [27, 49], in contrast to younger pelagic individuals [50–51]. As active swimmers preying on smaller benthic fish and invertebrates [50, 52], adult sablefish could be expected to react positively to increases of current velocity that lead to odor transport [53–54]. Sablefish have characteristically low-affinity hemoglobin, a characteristic which favors their active swimming, predatory life habits, but may result in reduced oxygen uptake within hypoxic waters [55]. Oxygen consumption in fish is directly related to the metabolic cost of locomotion [56], and the timing of sablefish activity within the hypoxic waters of Barkley Canyon during low flow conditions could be interpreted as a compromising strategy between search for prey and energy saving due to physiological limitations.

A diel displacement of the sablefish population within Barkley Canyon has been proposed, possibly in search of prey or to avoid predation by pelagic mammals, based on the phase difference between peaks in counts recorded from different fixed cameras [27]. Peaks in fish detection at consecutive temporal frames for nearby sampling sites can be used as an indication of populational movement [16, 57]. Nevertheless, the trajectory of the individuals in our study could not be followed with Wally. Thus, whether sablefish population moves from the hydrates site further up towards the canyon axis and back, on a daily basis, cannot be determined with 100% certainty. Further, coordinated research on that matter with the use of additional means (e.g. acoustic techniques), is needed to enlighten if the same population or distinct sub-populations are recorded at Barkley Canyon sites.

### 4.2 Activity rhythms of hagfish

Hagfish were found to be more associated with chemical variables such as chlorophyll concentration, rather than with flow. Their lifestyle is predominantly endobenthic, with animals spending most of their time resting coiled on the seabed (see Fig 2) or in self-built burrows [58–59]. Hagfish can opportunistically feed on several trophic levels [60], combining preying on small invertebrates and scavenging carcasses with the ability to take up dissolved organic matter *via* their skin [61–62]. While a high percentage of the organic matter within their diet can also originate from chemoautotrophs, many individuals’ isotopic signatures correspond to macroalgal sources (*Eptatretus sp*.; [60, 63–65]). No predation or scavenging activity was observed in the video recordings analyzed in this study. The robot did not film the nearby hydrate mound, where chemosynthetic bacteria formed white mats with seasonally varying surface coverage [3, 14], that may supply resuspended food to the surrounding environment. Chlorophyll rich organic matter settling to the seafloor could provide an additional food source and regulate the animal burrow emergence and activity rhythms on and above it.

Regarding their phase during the day-night cycle, hagfish have been reported as mainly nocturnal species both in laboratory conditions and *in situ* at various depths, from shallow waters down to depths of 300 m [32, 59, 66]. In contrast with these findings, two chronobiological studies conducted recently at Barkley Canyon reported arrhythmic activity for the hagfish [27, 35]. In our results, hagfish emerged mainly at crepuscular hours, at dawn, in a display of intraspecific plasticity of activity patterns [28]. Visual counts for hagfish in this study are higher than in the aforementioned studies, if the sampling period duration is taken into account, thus facilitating the detection of behavioral patterns. Hagfish are relatively sedentary animals [67] and the mobile nature of Wally enables the coverage of a greater area, providing a methodological advantage over fixed platforms. Although the underdeveloped eyes of species of the family Myxinidae can detect photoperiodic changes [68]), this inconsistency of Barkley Canyon case studies allows us to infer that at these depths environmental signals other than sunlight, such as the periodic chlorophyll influxes acting as an alternative food source, play an important role in synchronizing their circadian rhythms.

### 4.3 Activity rhythms of juvenile crabs

Our results suggest that the temporal pattern of juvenile crabs during the video transects is a combination of avoiding predation and physiological regulation by oxygen concentration. In the absence of sablefish from the study site during December of 2013, hagfish emerged as the major potential predator, known to prey on small benthic invertebrates, along with detritivory and a more opportunistic scavenging feeding behavior [69–71]. The waveforms of hagfish and crabs during that time have a semi-diurnal phase difference, inferring a possible avoidance behavior of the latter towards a potential predator. Minimizing predation risk through synchronization of diel activity is a common strategy in benthic decapod crustaceans [15–16]. In addition, this group appeared mostly during early night transects, when oxygen concentration was minimum. Benthic decapods of similar size have been observed to suppress their cryptic behavior under the effect of sharply induced hypoxia at a scale of hours (i.e. *Pilumnus spinifer*, *Pisidia longimana*; [72–73]), as well as in seasonally hypoxic conditions (*Munida quadrispina* small size class; [74]), which could explain their appearance in the field of view during minimum oxygen levels.

### 4.4 Methodology remarks

Extracting statistically meaningful results from relatively small faunal sample sizes was the principal methodological challenge we faced. Curve fitting based on non-linear least squares methods was used as an efficient alternative to the Lomb-Scargle periodogram analysis, since the latter can be sensitive to outliers at reduced sample sizes [75]. In contrast, although the non-linear least squares model may not provide a perfect fit from time to time (see Fig 3), the estimated wave period coefficients (i.e. a_4_) are indeed statistically significant (see Table 1).

The sampling frequency of 4 h was deemed adequate for a chronobiological study, as it complied with the Nyquist criterion for sampling. Indeed, the temporal windows of activity for any type of rhythm (diurnal, nocturnal, crepuscular) were not missed. Even though fewer samples were generated in comparison to fixed cabled observatory cameras with higher imaging rate, greater spatial coverage was achieved due to the mobility of the crawler, resulting in observed animal abundances comparable to previous studies. Nevertheless, a new generation of crawlers able to perform autonomous transects will provide even better temporal resolution and bigger datasets over longer period of time; a further tool to be used to gain a better understanding of deep-sea environments. The range of the crawler lights (~50 m) and the transect duration (< 20 min) restricted any possible attraction bias to fast swimming organisms. Sablefish that entered the field of view should be close enough, and they were not observed following the crawler during transects. Therefore, we are confident that results were not affected by attraction or double-counting. On a similar note, linear video-recording transects performed by the crawler simulate the visual census methods used by divers in shallower waters and can offer a solution to the ever-increasing need for volunteer citizen scientists [13, 76].

## 5. Conclusions

This study highlights the potential of the use of Internet Operated deep-sea Crawlers for generating high-quality biological and environmental datasets. Diel patterns were detected in the activity of the sablefish *A*. *fimbria*, the pacific hagfish *E*. *stoutii* and a group of juvenile crabs. These patterns were conditioned by different environmental parameters, depending on the ecology of each species. Flow magnitude was the variable mostly associated with swimmer sablefish, with the preference for mild flow conditions being a possible adaptation to the hypoxic waters of Barkley Canyon. On the other hand, the activity of species closely related to the seafloor was regulated by chemical properties such as chlorophyll and oxygen levels. Predator—prey relationships were also indicated in the abundance patterns, with the crabs possibly avoiding the hagfish and suppressing this avoidance behavior at times of minimum oxygen concentration.

## Supporting Information

S1 Fig Plots of residuals vs fitted values and histograms of residuals for the models presented in Fig 3. The plates correspond to a) and e) *A*. *fimbria* in June, b) and f) *A*. *fibria* in July, c) and g) *E*. *stoutii* in December and finally, d) and h) juvenile crabs in December.
(TIF)

S2 Fig Polar plots of flow conditions, depicting the magnitude (in m/s) and direction of water flow during the study period; a) June, b) July, and c) December. Each vector is an hourly averaged value.
(TIF)

S1 Table Hourly environmental data corresponding to the study period. (XLSX)

S2 Table Visual counts of studied species, together with concomitant environmental data averaged at 4 h frequency. (XLSX)

S3 Table Species and environmental variables scores for each RDA axis, per month. (XLSX)
