# Supplementary figures and images for: High-Frequency Patterns in the Abundance of Benthic Species near a Cold-Seep – An Internet Operated Vehicle Application
Source: PLoS One. 2016 Oct 12;11(10):e0163808. doi: 10.1371/journal.pone.0163808 (PMC5061432; doi:10.1371/journal.pone.0163808)

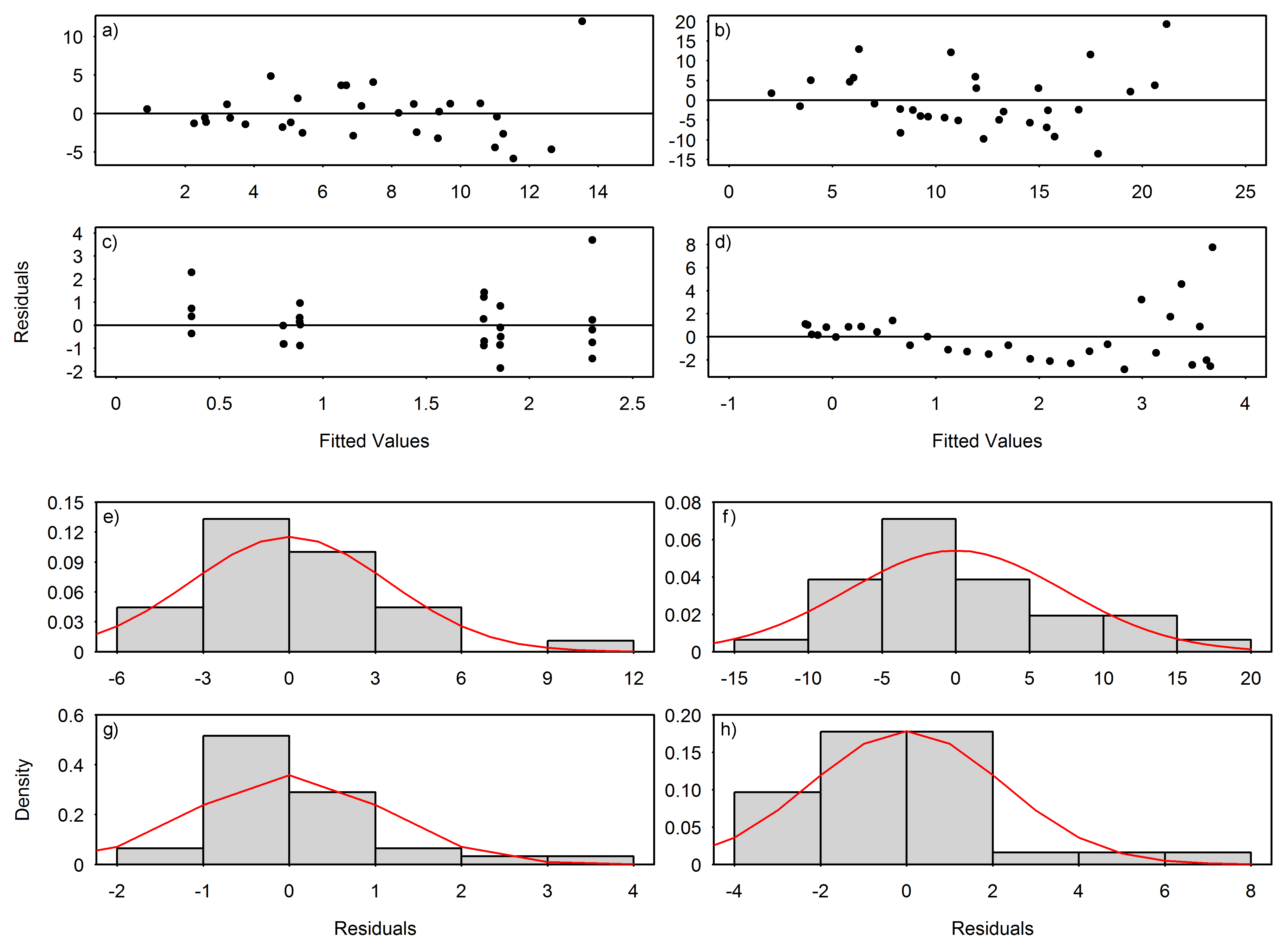

Supplement: S1 Fig — The plates correspond to a) and e) A. fimbria in June, b) and f) A. fibria in July, c) and g) E. stoutii in December and finally, d) and h) juvenile crabs in December. (TIF) [file pone.0163808.s001.tif]

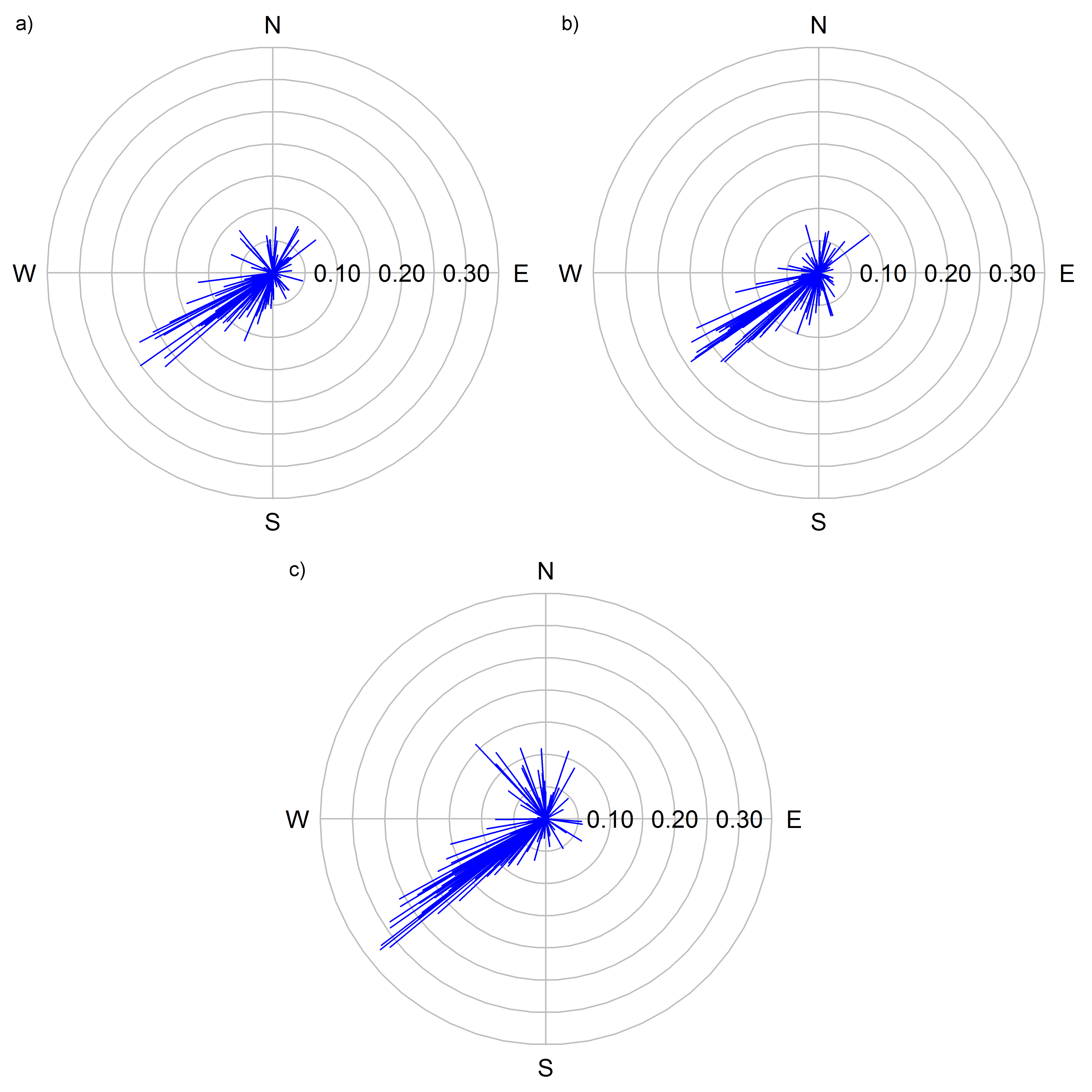

Supplement: S2 Fig — Polar plots of flow conditions, depicting the magnitude (in m/s) and direction of water flow during the study period; a) June, b) July, and c) December. Each vector is an hourly averaged value. (TIF) [file pone.0163808.s002.tif]
